# Use of rapid Model for End-Stage Liver Disease (MELD) increases for liver transplant registrant prioritization after MELD-Na and Share 35, an evaluation using data from the United Network for Organ Sharing

**DOI:** 10.1371/journal.pone.0223053

**Published:** 2019-10-03

**Authors:** Guy N. Brock, Kenneth Washburn, Michael R. Marvin

**Affiliations:** 1 Department of Biomedical Informatics and Center for Biostatistics, College of Medicine, The Ohio State University, Columbus, OH, United States of America; 2 Department of Surgery, Division of Transplantation Surgery, Wexner Medical Center, The Ohio State University, Columbus, OH, United States of America; 3 Center for Surgical Health Assessment, Research and Policy (SHARP), Wexner Medical Center, The Ohio State University, Columbus, OH, United States of America; 4 Department of Transplantation and Liver Surgery, Geisinger Medical Center, Danville, PA, United States of America; University of Colorado, UNITED STATES

## Abstract

The Model for End-Stage Liver Disease (MELD) score has been successfully used to prioritize patients on the United States liver transplant waiting list since its adoption in 2002. The United Network for Organ Sharing (UNOS)/Organ Procurement Transplantation Network (OPTN) allocation policy has evolved over the years, and notable recent changes include Share 35, inclusion of serum sodium in the MELD score, and a ‘delay and cap’ policy for hepatocellular carcinoma (HCC) patients. We explored the potential of a registrant’s change in 30-day MELD scores (ΔMELD_30_) to improve allocation both before and after these policy changes. Current MELD and ΔMELD_30_ were evaluated using cause-specific hazards models for waitlist dropout based on US liver transplant registrants added to the waitlist between 06/30/2003 and 6/30/2013. Two composite scores were constructed and then evaluated on UNOS data spanning the current policy era (01/02/2016 to 09/07/2018). Predictive accuracy was evaluated using the C-index for model discrimination and by comparing observed and predicted waitlist dropout probabilities for model calibration. After the change to MELD-Na, increased dropout associated with ΔMELD_30_ jumps is no longer evident at MELD scores below 30. However, the adoption of Share 35 has potentially resulted in discrepancies in waitlist dropout for patients with sharp MELD increases at higher MELD scores. Use of the ΔMELD_30_ to add additional points or serve as a potential tiebreaker for patients with rapid deterioration may extend the benefit of Share 35 to better include those in most critical need.

## Introduction

The Model for Endstage Liver Disease (MELD) scoring system has been highly successful in prioritizing patients on the waiting list for a liver transplant in the United States since its implementation in 2002 [1, 2]. However, even though the MELD score has been shown to be effective for a broad spectrum of liver disease [3], it is not without drawbacks [4]. As a result numerous modifications and enhancements to the MELD score have been proposed during the intervening period, including the incorporation of serum sodium [5, 6] and age [7] to the MELD score, reweighting of the MELD score components [8], and the change in serial MELD scores [9–11]. The latter approach, dubbed the delta MELD (ΔMELD) score, was conceived to address sudden or rapid deteriorations in disease status [9]. While proponents suggest that the ΔMELD is more effective than the standard MELD score for predicting waitlist mortality / dropout, other investigators have questioned its utility [12, 13].

The ΔMELD score was originally proposed by Merion *et al*. [9], who found that a ΔMELD of 5 or more within 30 days was a significant predictor of waitlist mortality even after accounting for serial MELD scores. However, follow-up studies determined that the ΔMELD was not an independent predictor of post-transplant mortality [12] or waitlist mortality [13]. In particular, Bambha *et al*. [13] demonstrated that the association between ΔMELD and waitlist mortality subsides once current MELD score and the number of serial measurements is taken into account. Nevertheless, subsequent studies continued to investigate utility of the ΔMELD score for predicting waitlist dropout and report positive findings. Specifically, Huo *et al*. [10] evaluated the ΔMELD score in 351 subjects and reported that the ΔMELD / month was more predictive (based on the C-index) of 6-month and 12-month waitlist mortality than either the standard MELD score or the Child-Turcotte-Pugh (CTP) score. However, as noted in the editorial by D’Amico [14], significant drawbacks of this study included exclusion of patients with only a single MELD score, lack of accounting for the number of MELD measurements, and comparison of ΔMELD with initial MELD score rather than serial MELD measurements. Another study [11] reported a positive association between the overall change in MELD from initial listing to the last recorded MELD score while on the waitlist with both waitlist and post-transplant mortality. However, since time between MELD measurements is not accounted for, this definition of the ΔMELD seemingly fails to differentiate between patients experiencing a rapid worsening of disease versus those with a more gradual decline.

All of aforementioned studies, irrespective of positive or negative findings, had a limited sample size (largest sample size of 1510 patients in [12]). That changed recently when Massie *et al*. [15] published a comprehensive study involving 69,643 registrants on the US liver transplant waitlist from 2002 to 2013. They evaluated the association between a MELD score spike (defined as a 30% or greater increase in MELD score over the previous 7 days) and waitlist mortality and found 2.3 times higher odds of 7-day mortality associated with a spike for registrants with a MELD score of 10, 4.0 times higher odds for a MELD score of 20, and 2.5 times higher odds for a MELD score of 30. Prediction of wait-list mortality was also improved with a model that incorporated both the MELD score and spike relative to the MELD score alone.

Since the Massie et al. study (REF), the United Network for Organ Sharing (UNOS)/Organ Procurement Transplantation Network (OPTN) implemented several important changes in liver organ allocation and registrant prioritization[16]. Share 35, initiated on June 18, 2013, prioritized transplantation for critically ill patients by offering donor organs to both local and regional registrants with MELD scores of 35 or higher. This has resulted in improved post-transplant mortality [17], center-level changes in organ offer acceptance rates [18], and potentially higher costs [19]. Another policy change in January 2016 was the incorporation of serum sodium into the MELD score calculation (called MELD-Na) for registrants with MELD > 11 [16, 20]. This change was based on a decade of evidence indicating that inclusion of serum sodium better predicts waitlist mortality and was projected to save up to 60 lives per year [5, 6, 21, 22]. Lastly, UNOS/OPTN implemented the ‘delay and cap’ policy changes in October 2015 to address the observed discrepancies in transplantation rates for patients with stage 2 hepatocellular carcinoma (HCC) [23–25]. This policy capped the MELD exception score for HCC patients at 34, and delayed receipt of exception points for HCC patients at initial listing for six months. The impact of these important changes in allocation policy on the relevance of the ΔMELD for liver waitlist prioritization has yet to be investigated.

In this study, we analyzed the UNOS data based on patients added to the waitlist between 06/30/2003 and 6/30/2013 (70,500 total registrants after accounting for exclusion criteria) and evaluated the association between ΔMELD scores with waitlist dropout, transplantation and post-transplant mortality. Baseline donor and registrant factors associated with ΔMELD increases were also evaluated. Two composite scores incorporating the ΔMELD were created, one which gave two additional MELD points for 30-day ΔMELD changes of 10 points or more (ΔMELD_30_ ≥ 10) and one which gave a variable number of points based on the patient’s MELD score. Predictive accuracy for models including current MELD and ΔMELD scores was assessed using the C-index for model discrimination and comparison of observed and predicted probabilities for model calibration. The two composite scores were then evaluated on UNOS data from 01/02/2016 to 09/07/2018, spanning the period after the recent changes in liver organ allocation policy. Differences in how ΔMELD scores were associated with patient dropout pre- and post-policy changes were evaluated.

## Materials and methods

### Data and study design

Data were obtained on all patients on the UNOS/OPTN liver transplant waitlist data as of 09/07/2018. Construction of composite scores incorporating the ΔMELD was based on registrants over the age of 18 years who were added to the waitlist between 06/30/2003 and 6/30/2013. Exception patients (e.g., HCC), Status 1, 1A, or 1B patients, patients with unknown status, and patients with only a single entry on the waitlist were also removed. Analysis was further restricted to active status observations for all patients. Data on waitlist registrants between 01/02/2016 to 09/07/2018 were filtered in a similar fashion and used to evaluate the composite scores incorporating the ΔMELD in the current policy era. The Institutional review board (IRB) at The Ohio State University determined the project did not qualify as human subjects research and did not require a formal IRB review.

### Outcomes and covariates

The primary outcome investigated was waitlist dropout, with secondary outcomes including transplantation and post-transplant mortality. For waitlist data, patient dropout was defined as any patient whose last follow-up record on the waitlist was scored as medically unsuitable, too sick to transplant or died. Patients who were transplanted at any facility for any reason were considered as transplanted. All other patients were censored at their last waitlist follow-up record. For cause-specific hazards models, transplantation was considered a censoring event when analyzing waitlist dropout and conversely dropout was considered a censoring event for transplantation. For changes in MELD score we focused on the 30-day ΔMELD score, abbreviated as ΔMELD_30_, since we felt this represented a clinically manageable period of time for which to evaluate changes in practice. For consecutive observations on a patient that are within 30 days of each other, ΔMELD_30_ was defined as the difference between the largest MELD score within the 30-day window and the current MELD score (similar to Bambha *et al*. [13]). For consecutive observations greater than 30 days apart, ΔMELD_30_ was defined as the difference between the current and prior MELD score divided by the number of 30-day intervals. In addition to the ΔMELD_30_ score, we used pre-determined thresholds based on the prior literature of ΔMELD_30_ ≥ 5 and ≥ 10 as well as a 30% increase in MELD score [9, 11, 13, 15]. To allow for changes in MELD scores exceeding 40 points, ΔMELD_30_ was based on uncapped MELD scores. All patients had an initial ΔMELD_30_ score of zero. The number of lab measurements within the 30-day window was recorded and included in the multivariable model as a covariate [13]. Other covariates evaluated included patient age, ethnicity, body mass index (BMI), hepatitis C virus (HCV) serostatus, hepatitis B virus (HBV) core status, diabetes, coronary artery disease (CAD), share type, donor age, and serum sodium.

### Statistical methods

Cause-specific hazard (CSH) Cox regression models (which censor for competing events) were used to model the association between ΔMELD scores and the time to waitlist dropout. Since the CSH model censors for transplantation when investigating waitlist dropout, the associations are uninfluenced by transplantation. This model is most closely tied to the underlying biology and is informative in a comparison of scores ‘starting from scratch’, i.e. in a hypothetical situation where patients are not currently prioritized on the basis of the MELD score.

Association between ΔMELD scores and waitlist dropout were adjusted for current MELD and the number of measurements within the 30 day observational window using multivariable models. Additionally, we assessed the potentially differential effect of ΔMELD scores on waitlist dropout by fitting models including interaction terms between ΔMELD and strata of MELD scores 6–10, 11–15, 16–20, 21–25, 26–30, 31–35, and 36–40. Incorporation of ΔMELD into a composite MELD score was done by equating the linear predictors from a model including MELD score and a model including both MELD and ΔMELD, similar to what we have done previously for hepatocellular carcinoma patients on the waitlist [26]. Similar models were fitted to evaluate the association between ΔMELD and transplantation. Association between ΔMELD and post-transplant patient mortality was evaluated using Cox models. For predictive accuracy we evaluated both discriminatory power (Harrell’s C-index [27]) and model calibration (by comparing observed and predicted one, three, and six month waitlist dropout). The C-index estimates the probability that, for a randomly selected pair of individuals, the individual with the higher risk score (e.g., MELD or composite MELD / ΔMELD score) has the shorter actual event time (time to waitlist dropout). Observed dropout was estimated using cumulative incidence functions (which treat transplant as a competing risk) using time since each subject first obtained a given MELD score. Analyses were conducted using R version 3.4.1 [28] with the *survival* package [29] for Cox models.

## Results

A total of 70,500 patients met our inclusion criteria during the time frame between 06/30/2003 and 06/30/2013, with an average number of 10.6 waiting list records per patient (675,018 total records). Forty-nine percent of registrants (34,566) were transplanted, 23.6% (16,625 registrants) were removed from the waitlist due to death (10,522 registrants, 16.6%) or being too sick for transplant (6,103 registrants, 8.7%), 4% (2,843 registrants) were removed due to improvement, and 23.4% (16,466 registrants) were still on the waitlist at the end of the timeframe. Ninety-one percent (614,821/675,018) of the ΔMELD_30_ scores fell between -2 and 7, with a median of zero and a mean of 0.98. While most (299,761/675,018; 44.4%) of the ΔMELD_30_ scores were zero, 10.4% (70,500/675,018) were due to being the initial observation on a subject. Similarly, many of the observations were the only record within 30 days (278,944/675,018; 41.3%), while 29.4% (198,213/675,018) had two records and 10.1% (68,004/675,018) had three. The median time between consecutive measurements was 23 days, with 90% of follow-up measurements occurring within 3 months. The percentage of observations having a ΔMELD_30_ of 5 or more was 8.9% (60,401/675,018), while 3.2% (44,768/675,018) of observations had a ΔMELD_30_ of 10 or more and 6.6% (44,768/675,018) of observations had a 30% increase in MELD score over 30 days. The correlation between ΔMELD_30_, MELD, number of measurements within the 30-day window and time between consecutive measurements is given in supplementary S1 Table. As expected, the ΔMELD_30_ is strongly correlated with current MELD score and the number of measurements within 30 days, and negatively correlated with the time since the last measurement.

**Fig 1:**
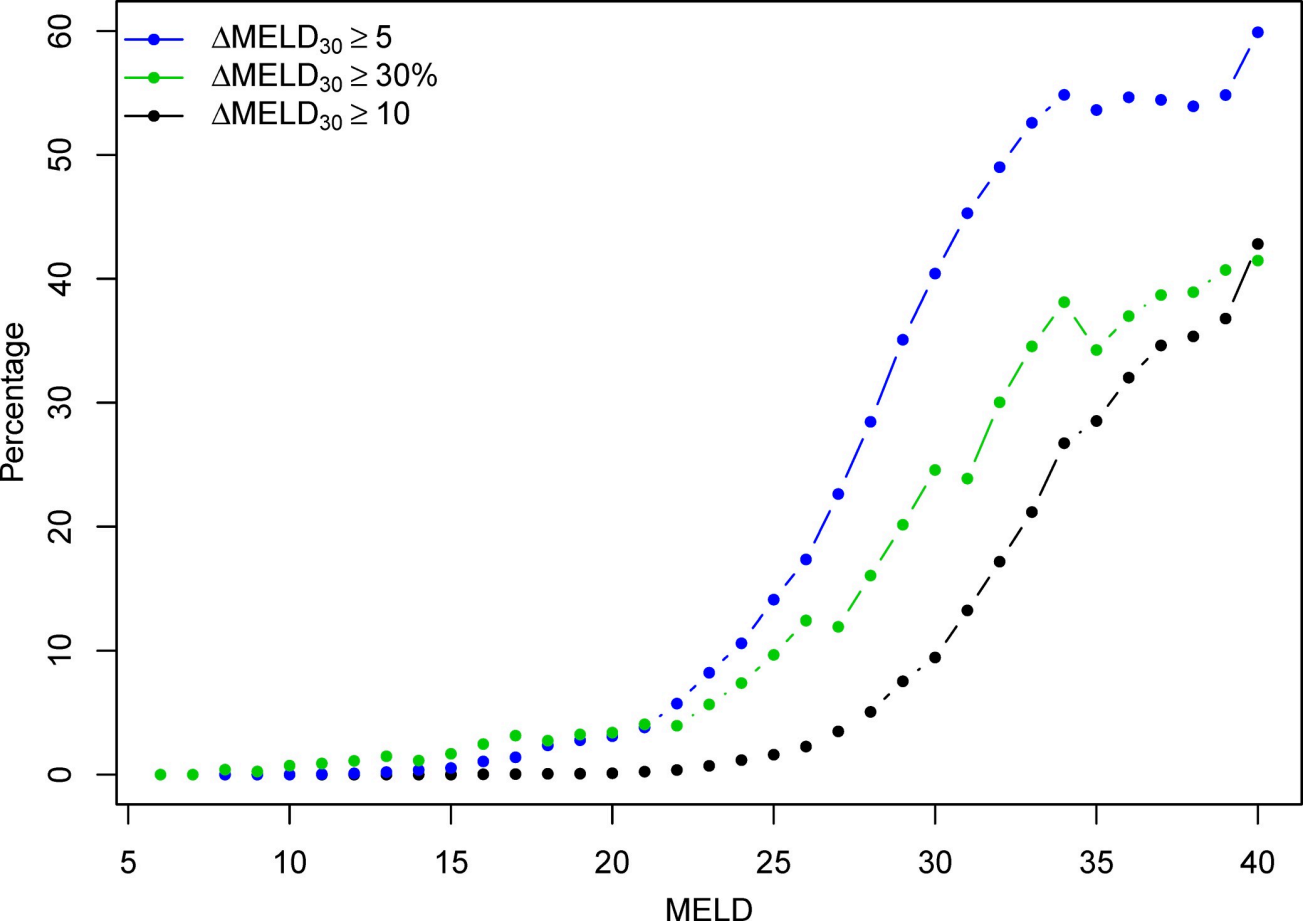
Percentage of observations with ΔMELD_30_ ≥ 10, ΔMELD_30_ ≥ 5, and ΔMELD_30_ of 30% or more at each MELD score.

**Fig 2:**
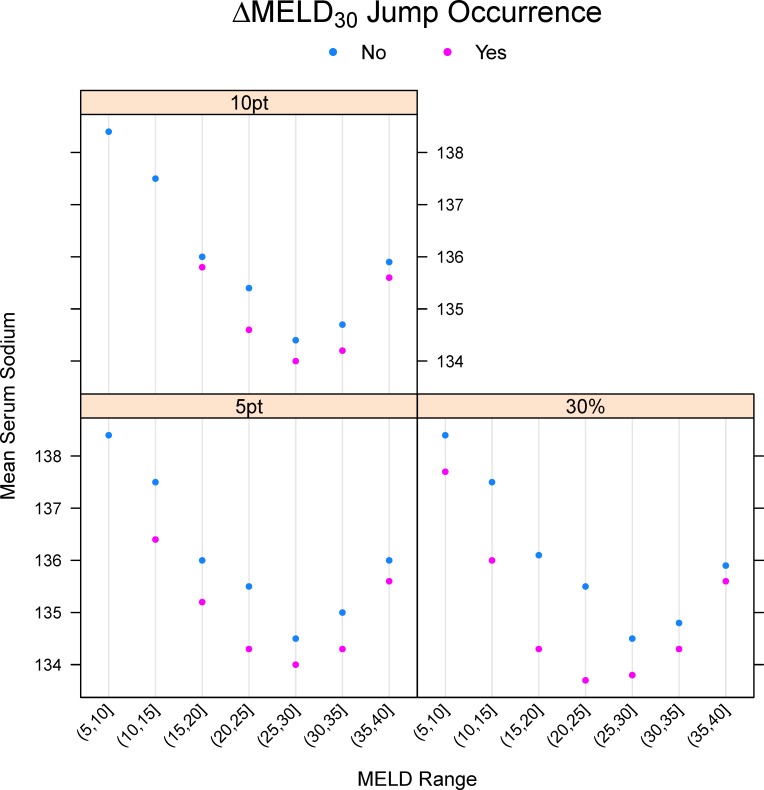
Mean serum sodium for patients with and without occurrence of a ΔMELD_30_ jump.

Fig 1 displays the percentage of observations at each MELD score with ΔMELD_30_ ≥ 10, ΔMELD_30_ ≥ 5, and ΔMELD_30_ ≥ 30%. ΔMELD_30_ changes of those magnitudes were relatively infrequent at MELD scores of 20 or less (3.4% of observations or fewer). For MELD scores between 20 and 30, ΔMELD_30_ jumps of 10 points or more were still relatively infrequent while changes of 5 points or more rose to 40% of observations at MELD scores of 30. At MELD scores of 35 or more, at least 30% of the observations had ΔMELD_30_ ≥ 10 and 50% of observations had ΔMELD_30_ ≥ 5. To investigate which patient factors were associated with drastic changes in MELD scores, Table 1 displays the characteristics of patients experiencing a ΔMELD_30_ ≥ 10 (8,322 patients, 11.8%) compared to those who did not (62,178 patients, 88.2%). Patients experiencing ΔMELD_30_ ≥ 10 were more commonly Hispanic and less frequently White, were more likely to be diagnosed with Type C cirrhosis at registration and less likely to be diagnosed with alcoholic cirrhosis, had a smaller percentage of national shares and greater percentage of regional shares, and had a slightly lower donor age. These patients also had a higher percentage of positive HCV serostatus and HBV core status and a greater percentage were hospitalized at registration, but the percentage of missing values for these variables exceeded 50% so no firm conclusions can be made. Patient age, diabetes, CAD, and BMI were all not statistically significant. Mean serum sodium was higher for patients experiencing a ΔMELD_30_ jump (p < 0.001), with the largest difference occurring in patients with MELD scores between 10 and 25 (Fig 2).

**Table 1:** Baseline registrant and donor organ characteristics for registrants stratified by ΔMELD_30_ ≥ 10 status.

		ΔMELD_30_ ≥ 10		
	Level	No	Yes	p-value^a^
Number		62,178	8,322	
Registrant Age (mean (sd))		52.80 (10.45)	52.76 (10.19)	0.718
Registrant Ethnicity (%)	White	45,512 (73.2)	5,655 (68.0)	<0.001
	Black	5,364 (8.6)	688 (8.3)	
	Hispanic	8,755 (14.1)	1,613 (19.4)	
	Asian	1,862 (3.0)	255 (3.1)	
	Other	685 (1.1)	111 (1.3)	
Registrant Diabetes (%)	No	45,754 (78.7)	6,139 (78.3)	0.45
	Type I/II	12,410 (21.3)	1,703 (21.7)	
	Missing	4,014	480	
Registrant CAD^b^ (%)	No	7,048 (97.6)	715 (97.9)	0.685
	Yes	171 (2.4)	15 (2.1)	
	Missing	54,959	7,592	
Registrant BMI (mean (sd))		28.58 (5.88)	28.70 (5.93)	0.199
	Missing	32,759	3,294	
Registrant HBV core (%)	N	20,653 (81.0)	3,625 (79.7)	0.035
	P	4,844 (19.0)	926 (20.3)	
	Missing	36,681	3,771	
Registrant HCV serostatus (%)	N	16,167 (61.1)	2,593 (55.3)	<0.001
	P	10,273 (38.9)	2,097 (44.7)	
	Missing	35,738	3,632	
Diagnosis at Registration (%)	Cirrhosis: Type C	17,680 (28.4)	2,658 (31.9)	<0.001
	Alcoholic Cirrhosis	11,579 (18.6)	1,170 (14.1)	
	Liver (NASH)	4,743 (7.6)	658 (7.9)	
	Fatty Cirrhosis: Cryptogenic (Idiopathic)	4,379 (7.0)	521 (6.3)	
	Alcoholic Cirrhosis With Hepatitis C	4,121 (6.6)	581 (7.0)	
	Primary Biliary Cirrhosis	2,206 (3.5)	380 (4.6)	
	Cirrhosis: Autoimmune	2,092 (3.4)	294 (3.5)	
	PSC^c^: Ulcerative Colitis	1,387 (2.2)	225 (2.7)	
	Cirrhosis: Type B- HBSAG+	1,303 (2.1)	145 (1.7)	
	PSC: No Bowel Disease	920 (1.5)	143 (1.7)	
	Other	11,768 (18.9)	1,547 (18.6)	
Medical Condition at Registration (%)	Hospitalized, not in ICU^d^	2,399 (11.4)	371 (18.5)	<0.001
	In ICU	1,122 (5.3)	158 (7.9)	
	Not Hospitalized	17,519 (83.3)	1,480 (73.7)	
	Missing	41,138	6,313	
Share Type (%)	Local	21,689 (73.5)	3,757 (74.3)	<0.001
	Regional	5,942 (20.1)	1,147 (22.7)	
	National	1,879 (6.4)	152 (3.0)	
	Missing	32,668	3,266	
Donor Age (mean (sd))		40.79 (16.89)	39.93 (15.90)	0.001
	Missing	32,668	3,266	

^a^ p-values and percentages were calculated using only non-missing data. T-tests were used for continuous data and chi-squared tests for categorical data.

^b^ CAD = Coronary artery disease

^c^ PSC = Primary sclerosing cholangitis

^d^ ICU = Intensive care unit

Hazard ratios (HRs) and predictive accuracy (C-indexes) for univariable and multivariable CSH models for patient dropout from the waitlist are given in Table 2. The ΔMELD_30_ and all its derivatives are strongly associated with the cause-specific waitlist dropout hazard in univariable models, but after inclusion of current MELD the strength of these associations falls dramatically while the hazard ratio (HR) for current MELD score remains relatively unchanged. In addition, the predictive ability (C-index) for each of the multivariable CSH models is nearly identical to that for current MELD score alone. Multivariable models, (which additionally included serum sodium, patient ethnicity, patient diagnosis at time of registration, and number of records within the past 30 days on the waitlist) further reduced the magnitude of both MELD and ΔMELD_30_ though both remained strongly statistically significant. HCV serostatus, HBV core status, and medical condition at registration were excluded from the multivariable models due to the large percentage of missing values. The interaction between MELD and ΔMELD_30_ ≥ 10 was highly significant in both the univariable and multivariable models, and indicated a decrease in magnitude of effect of ΔMELD_30_ ≥ 10 with increasing MELD score.

**Table 2:** Univariable and multivariable CSH models for waitlist dropout using MELD and ΔMELD_30_.

	Unadjusted Models			Adjusted (multivariable) Models^a^		
Variable	exp(β)^b^	95% CI^c^	C-index (SE)^d^	exp(β)	95% CI^c^	C-index (SE)^d^
**Singleton MELD / ΔMELD**						
Current MELD	1.2	(1.19, 1.2)	0.819 (0.003)	1.17	(1.17, 1.17)	0.827 (0.003)
ΔMELD_30_	1.23	(1.23, 1.24)	0.669 (0.002)	1.16	(1.16, 1.17)	0.768 (0.003)
ΔMELD_30_ ≥ 5	15.52	(14.94, 16.12)	0.614 (7e-04)	5.25	(5.02, 5.5)	0.77 (0.003)
ΔMELD_30_ ≥ 30%	9.85	(9.44, 10.26)	0.576 (6e-04)	6.95	(6.65, 7.26)	0.782 (0.003)
ΔMELD_30_ ≥ 10	32	(30.46, 33.61)	0.562 (4e-04)	9.12	(8.61, 9.66)	0.768 (0.003)
**Combined MELD / ΔMELD Models**						
1. Current MELD	1.18	(1.18, 1.18)	0.819 (0.003)	1.16	(1.16, 1.16)	0.827 (0.003)
ΔMELD_30_	1.03	(1.03, 1.04)		1.02	(1.02, 1.03)	
2. Current MELD	1.18	(1.18, 1.18)	0.819 (0.003)	1.16	(1.16, 1.16)	0.827 (0.003)
ΔMELD_30_ ≥ 5	1.57	(1.5, 1.65)		1.39	(1.31, 1.46)	
3. Current MELD	1.18	(1.18, 1.19)	0.82 (0.003)	1.16	(1.16, 1.17)	0.827 (0.003)
ΔMELD30 ≥ 30%	1.55	(1.48, 1.63)		1.4	(1.33, 1.48)	
4. Current MELD	1.19	(1.18, 1.19)	0.819 (0.003)	1.16	(1.16, 1.17)	0.827 (0.003)
ΔMELD_30_ ≥ 10	1.47	(1.39, 1.56)		1.3	(1.22, 1.38)	
5. Current MELD	1.19	(1.19, 1.19)	0.819 (0.003)	1.17	(1.16, 1.17)	0.827 (0.003)
ΔMELD_30_ ≥ 10	7.1	(4.75, 10.61)		6.77	(4.43, 10.34)	
MELD*ΔMELD_30_ ≥ 10	0.96	(0.95, 0.97)		0.96	(0.95, 0.97)	

^a^ Adjusted (multivariable) models included registrant ethnicity (categorized as White, Black, Hispanic, Asian and Other), diagnosis at registration (Cirrhosis Type C, Alcoholic Cirrhosis, or Other), serum sodium, and number of records within the past 30 days on the waitlist, in addition to the variables shown in the table.

^b^ exp(β) is equivalent to the hazard ratio (HR) for all models except the interaction model (combined Model #5), where the interpretation of the coefficients is more complex.

^c^ All p-values were < 0.0001

^d^ C-index applies to the entire model

To better illustrate the interaction between MELD and ΔMELD_30_ scores, we fit models for waitlist dropout incorporating interaction between ΔMELD_30_ ≥ 10 and different MELD strata (Table 3). Models were not adjusted for additional covariates. The lowest MELD stratum was 16–20 to allow 10 point increases in ΔMELD_30_. The interaction between ΔMELD_30_ and current MELD strata was highly significant (p<0.001), reflected by the decreasing HR associated with ΔMELD_30_ ≥ 10 as the MELD strata increased (from 3.81 in 16–20 stratum to 1.52 in 36–40 stratum). We also investigated whether ΔMELD_30_ scores were associated with increased rates of transplantation using the same interaction model but with transplantation as the outcome (Table 3). The interaction term in the CSH model between ΔMELD_30_ ≥ 10 and MELD strata was statistically significant (p = 0.03), with transplantation HRs for ΔMELD_30_ ≥ 10 ranging from 3.89 for MELD scores of 16–20 to 1.65 for MELD scores of 36–40. In every case the HRs were significantly above one, potentially reflecting that physicians are currently incorporating rapid changes in MELD score for prioritizing patients for transplantation.

**Table 3:** Hazard ratios for waitlist dropout and transplantation for ΔMELD_30_ ≥ 10 vs < 10, stratified by current MELD. Models were not adjusted for additional covariates.

	MELD Strata	HR (ΔMELD_30_ ≥ 10 vs < 10)	95% CI	P-value
Waitlist Dropout	16–20	3.81	(1.58, 9.15)	0.003
	21–25	3.35	(2.53, 4.43)	<0.001
	26–30	2.35	(1.97, 2.80)	<0.001
	31–35	1.77	(1.56, 2.01)	<0.001
	36–40	1.52	(1.41, 1.64)	<0.001
Transplantation				
	16–20	3.89	(2.15, 7.02)	<0.001
	21–25	1.88	(1.49, 2.38)	<0.001
	26–30	1.75	(1.56, 1.97)	<0.001
	31–35	1.57	(1.45, 1.69)	<0.001
	36–40	1.65	(1.56, 1.74)	<0.001

To incorporate ΔMELD into the current MELD scoring system, we equated the linear predictors from a model for waitlist dropout including MELD score and ΔMELD_30_ to a model for waitlist dropout including current MELD score alone. We selected two models, the MELD + ΔMELD_30_ ≥ 10 model (Model #4 in Table 2) and the model which additionally includes the interaction between those two terms (Model #5 in Table 2) for comparison since these reflect the most drastic change in ΔMELD among the models we evaluated. For Model #4, this results in equating 0.178*MELD to 0.171*MELD + 0.386*I(ΔMELD_30_ ≥ 10), where I() is the indicator function and is one if ΔMELD_30_ ≥ 10 and zero otherwise. Then the new composite MELD score is MELD_NEW_ = 0.171/0.178*MELD_CURRENT_ + 0.386/0.178*I(ΔMELD_30_ ≥ 10) = 0.96*MELD_CURRENT_ + 2.17*I(ΔMELD_30_ ≥ 10). Rounding the coefficients to integers then gives MELD_NEW_ = MELD_CURRENT_ + 2*I(ΔMELD_30_ ≥ 10). In other words, the new composite MELD score would be the current MELD score plus two additional points if the MELD score increased by 10 or more points within the last 30 days. The approach for Model #5 is identical but the resulting increase in MELD points will depend on the MELD score given the inclusion of the interaction term (see Table 4).

**Table 4:** Modified MELD score which accounts for increased risk in waitlist dropout for patients experiencing ΔMELD_30_ ≥ 10.

MELD Score Range	Increase in MELD score to account for ΔMELD_30_ ≥ 10 dropout risk
16	7
17–20	6
21–23	5
24–27	4
28–30	3
31–34	2
35–38	1
39–40	0

Results are based on the interaction model between MELD and ΔMELD_30_ ≥ 10 (Model #5 in Table 2). There is a larger difference between MELD and modified MELD for patients with lower MELD scores compared to those with higher MELD scores, a reflection of the interaction term. MELD scores below 16 are not shown since patients below that score could not have experienced a 10 point jump.

**Fig 3:**
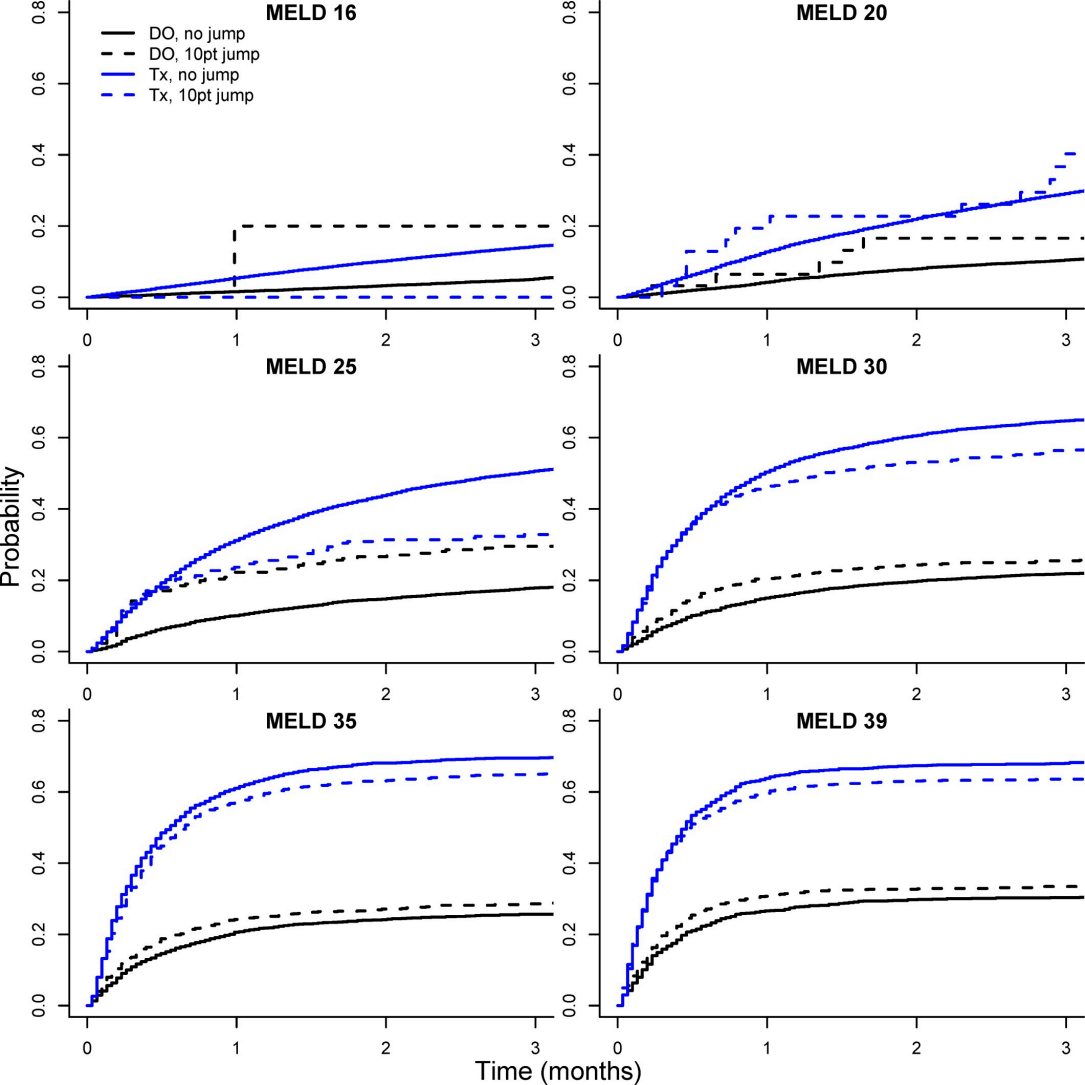
Cumulative incidence curves for waitlist dropout (DO) and transplantation (Tx). Cumulative incidence curves for waitlist dropout (black lines) and transplantation (blue lines) for patients with (dashed line) and without (solid line) a ΔMELD_30_ ≥ 10 jump at various MELD scores. Based on UNOS/OPTN data for registrants added to the liver transplant waitlist between 06/30/2003 and 6/30/2013.

**Fig 4:**
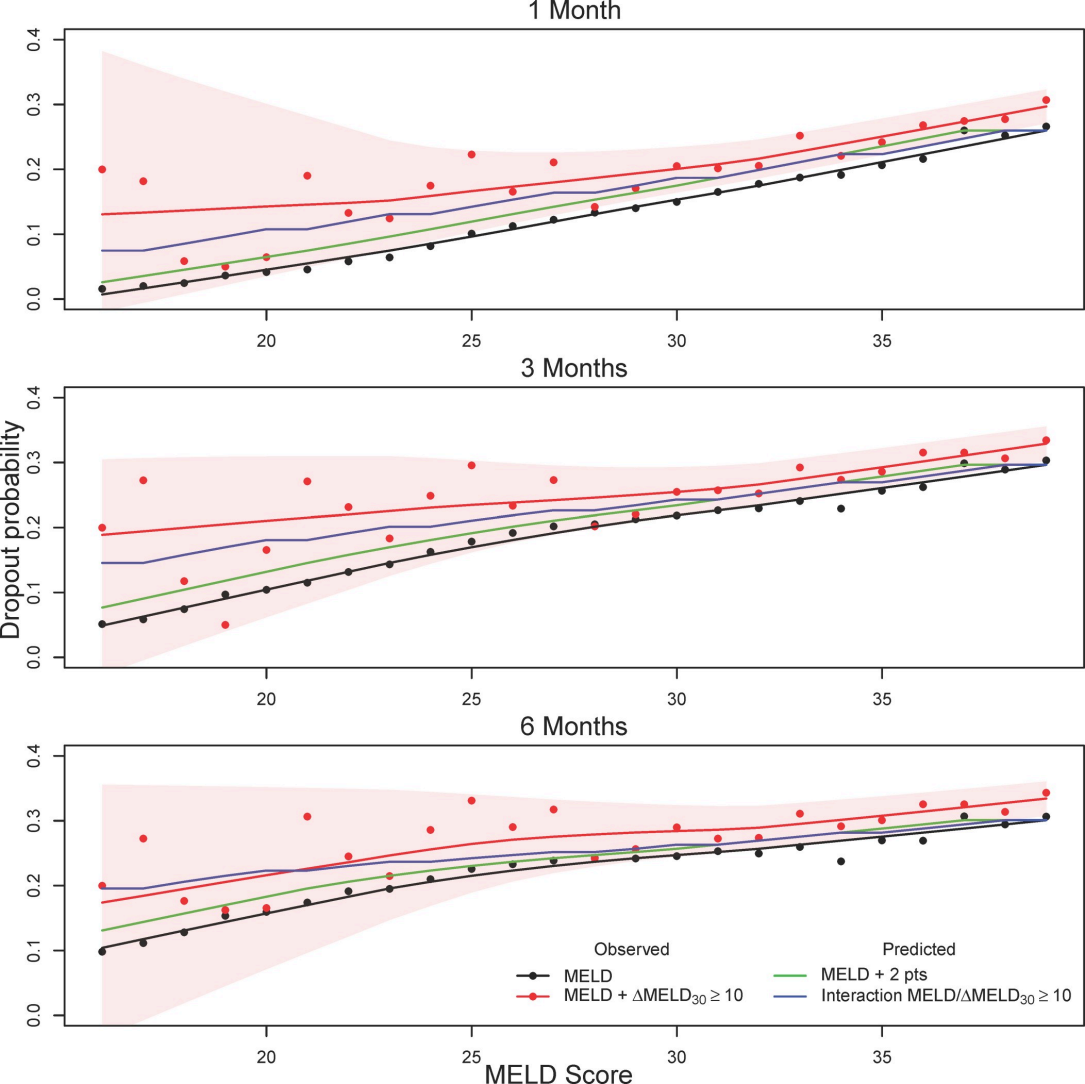
Observed versus predicted one, three, and six month waitlist dropout. Observed versus predicted one, three, and six month waitlist dropout from the first time a given MELD score is obtained for patients experiencing a ΔMELD_30_ ≥ 10 jump. Red points and smoothed red lines are observed probabilities for patients experiencing a ΔMELD_30_ ≥ 10 jump, while black points and smoothed black lines are observed probabilities for patients not experiencing a ΔMELD_30_ ≥ 10 jump. Red shaded regions are pointwise 95% confidence intervals for patients with ΔMELD_30_ ≥ 10, where the upper and lower limits have been smoothed for better presentation. The green line indicates the predicted dropout for actual MELD plus two points (based on Model #4 in Table 2), while the blue line indicates the predicted dropout for the MELD / ΔMELD_30_ ≥ 10 interaction model (Model #5 in Table 2, with increase in MELD score given in Table 4). Based on UNOS/OPTN data for registrants added to the liver transplant waitlist between 06/30/2003 and 6/30/2013.

The predictive accuracy based on the C-index for these two models are nearly identical to the standard MELD score (Table 2). However, this is perhaps not surprising given that ΔMELD_30_ ≥ 10 was a relatively infrequent occurrence (only 3.2% of total observations). To better visualize the difference in waitlist dropout associated with ΔMELD_30_ ≥ 10 we compared observed and predicted waitlist dropout probabilities for patients experiencing a ΔMELD_30_ ≥ 10 jump from the first time they obtained a given MELD score. Fig 3 displays cumulative incidence curves for waitlist dropout (DO) and transplantation (Tx) for patients with (dashed line) and without (solid line) a ΔMELD_30_ ≥ 10 jump at various MELD scores. In general, patients with the ΔMELD_30_ ≥ 10 jump had higher dropout probability and corresponding lower transplant probability. Fig 4 further compares observed dropout probabilities for patients with the ΔMELD_30_ ≥ 10 jumps with those predicted by modeling. Predicted dropout for the MELD / ΔMELD_30_ ≥ 10 interaction model (model #5 in Table 2, blue line) is much closer to observed dropout for patients with ΔMELD_30_ ≥ 10 (red line and points) compared to observed dropout for MELD score alone (black line and points). The green line indicates predicted dropout for MELD plus two points (based on Model #4 in Table 2). This line underestimates the observed ΔMELD_30_ ≥ 10 dropout for lower MELD scores but is fairly close to the blue line for the interaction model from MELD scores of 24 onwards. However, there is considerable variability in the estimate of ΔMELD_30_ ≥ 10 dropout (red shaded region), particularly at MELD scores below 25 where ΔMELD_30_ ≥ 10 jumps are infrequent (see Fig 1).

**Fig 5:**
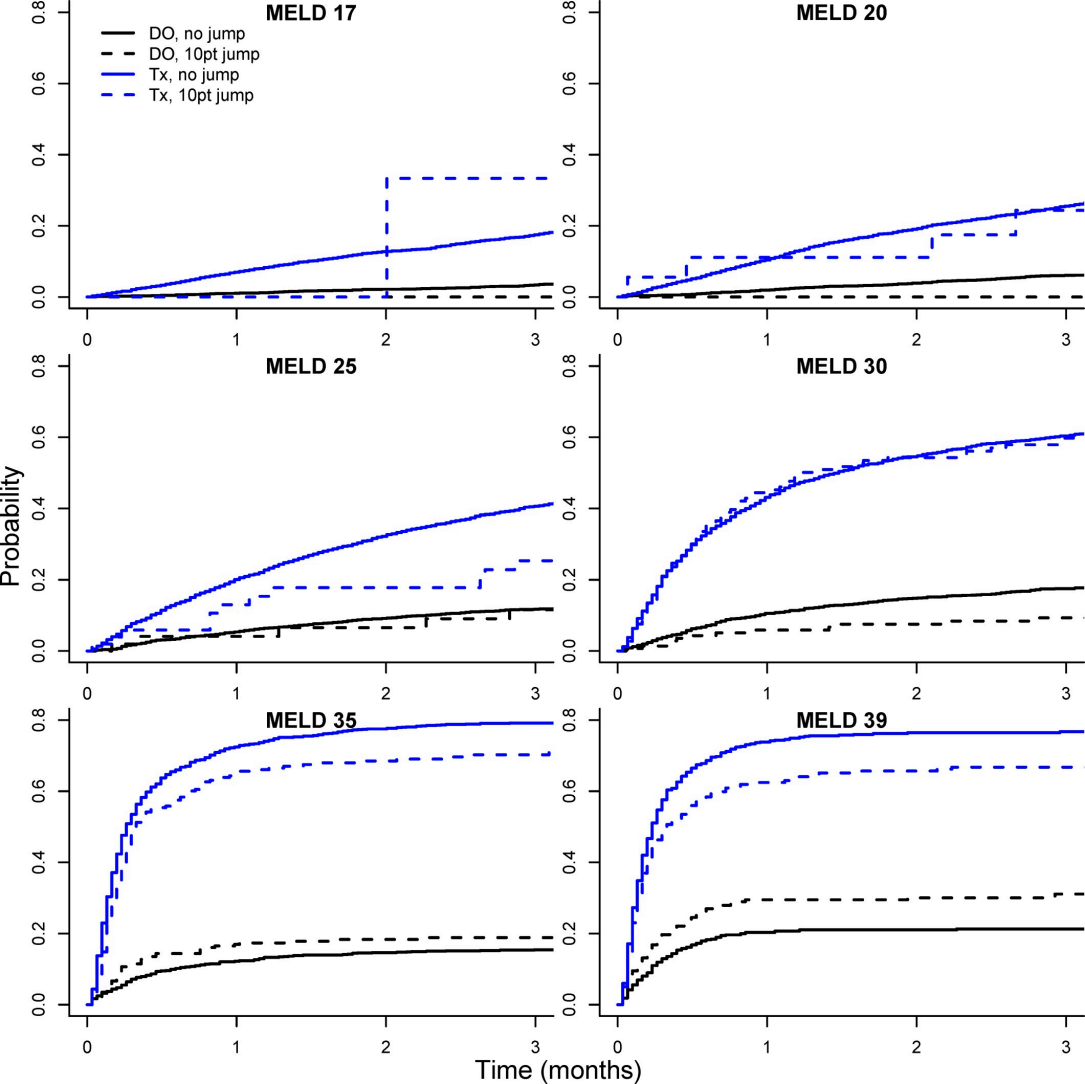
Cumulative incidence curves for waitlist dropout (DO) and transplantation (Tx) in data post MELD-Na and Share 35. Cumulative incidence curves for waitlist dropout (black lines) and transplantation (blue lines) for patients with (dashed line) and without (solid line) a ΔMELD_30_ ≥ 10 jump at various MELD scores. Based on UNOS/OPTN data for registrants on the liver transplant waitlist between 01/02/2016 to 09/07/2018.

**Fig 6:**
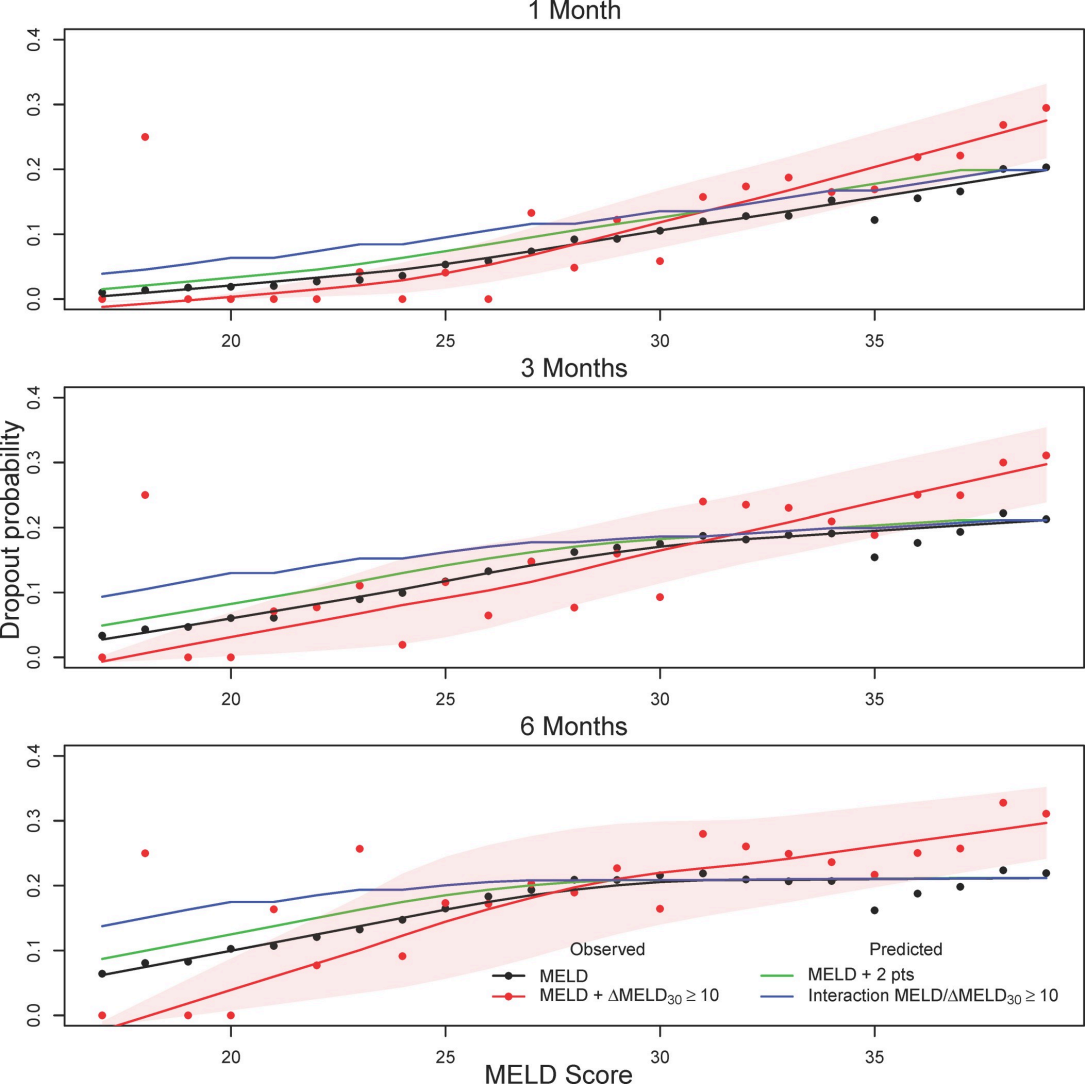
Observed versus predicted one, three, and six month waitlist dropout in data post MELD-Na and Share 35. Observed versus predicted one, three, and six month waitlist dropout from the first time a given MELD score is obtained for patients experiencing a ΔMELD_30_ ≥ 10 jump. Red points and smoothed red lines are observed probabilities for patients experiencing a ΔMELD_30_ ≥ 10 jump, while black points and smoothed black lines are observed probabilities for patients not experiencing a ΔMELD_30_ ≥ 10 jump. Red shaded regions are pointwise 95% confidence intervals for patients with ΔMELD_30_ ≥ 10, where the upper and lower limits have been smoothed for better presentation. The green line indicates the predicted dropout for actual MELD plus two points (based on Model #4 in Table 2), while the blue line indicates the predicted dropout for the MELD / ΔMELD_30_ ≥ 10 interaction model (Model #5 in Table 2, with increase in MELD score given in Table 4). Based on UNOS/OPTN data for registrants on the liver transplant waitlist between 01/02/2016 to 09/07/2018.

Fig 5 displays cumulative incidence curves for waitlist dropout (DO) and transplantation (Tx) in the cohort spanning the period after the recent changes in liver organ allocation policy (01/02/2016 to 09/07/2018). In contrast to Fig 3, patients with ΔMELD_30_ ≥ 10 jumps no longer have higher dropout probability and lower transplant probability except at the higher MELD scores (35 and 39). This is further demonstrated in Fig 6, where the dropout probability for patients with ΔMELD_30_ ≥ 10 (red line and points) is at or below the line for MELD score alone (black line and points) until MELD scores above 30. This is quite different from Fig 4, which demonstrates substantial difference in dropout probability at lower MELD scores between patients with and without ΔMELD_30_ ≥ 10 jumps which diminishes with increasing MELD score.

## Discussion

In this study, we performed a detailed evaluation of the utility of the 30-day delta MELD score (ΔMELD_30_) for predicting both waitlist dropout and post-transplant mortality among liver transplant registrants in the United States. Using registrants added to the waitlist between 06/30/2003 and 6/30/2013, we constructed two composite registrant prioritization scores that combined the ΔMELD and standard MELD score. Similar to Massie *et al*. [15], we found the ΔMELD score to be a significant independent predictor of waitlist dropout after accounting for current MELD score. Our mapping of ΔMELD_30_ ≥ 10 occurrences to additional MELD points (Table 4) was also similar to what they obtained for a 30% increase in 7-day ΔMELD (c.f. Table 3 in their paper). However, while accuracy for waitlist dropout of patients with ΔMELD_30_ ≥ 10 changes was improved the overall discriminatory ability as measured by the C-index was not improved by the ΔMELD_30_.

We further evaluated use of the ΔMELD_30_ for registrants added to the waitlist after three important policy changes: Share 35, ‘delay and cap’ policy for HCC patients, and a switch to the MELD-Na score for registrants with MELD > 11 [16]. Calibration plots based on this cohort differed substantially from those based on data before the policy changes (c.f. Fig 4 and Fig 6). Prior to the policy changes, differences patients experiencing a ΔMELD_30_ ≥ 10 jump had higher dropout in the lower to middle MELD score ranges (15–25). After the policy changes these differences were no longer present. Since ΔMELD_30_ jumps correlated with hyponatremia, adoption of the MELD-NA has seemingly eliminated discrepancies in dropout probability associated with sharp MELD increases at the lower range. This corroborates with prior studies which have shown that serum sodium is particularly relevant to waitlist mortality risk for patients in the lower MELD score range [6].

In contrast, patients experiencing a ΔMELD_30_ ≥ 10 jump at the higher MELD score range (30 and above) seem to have greater discrepancy in dropout probability in the current policy era. Share 35 makes organs more readily available to registrants with MELD scores of 35 or more, and likely benefits patients who have been at the higher MELD scores for longer periods of time. However, patients with sudden increases at the higher MELD score range still experience high dropout rates and appear to have benefitted less from the policy change (c.f. cumulative incidence curves in Fig 3 and Fig 5). Models incorporating the ΔMELD built on data prior to these policy changes do not fully capture the differences in dropout at the higher MELD range, as only a few additional points were given for ΔMELD_30_ ≥ 10 jumps for MELD scores above 30 (Table 4).

We evaluated a number of patient characteristics at baseline to determine whether certain factors predisposed patients to sharp increases in MELD scores. There was a significant inverse association between serum sodium levels and patients with ΔMELD_30_ ≥ 10 jumps. While positive HCV serostatus and hospitalized at registration were higher among patients experiencing a ΔMELD_30_ ≥ 10 jump, the percentage of missing values for these variables exceeded 50%. Patients experiencing a ΔMELD_30_ ≥ 10 jump were more likely to be diagnosed with Type C cirrhosis and less likely to be diagnosed with alcoholic cirrhosis, and were more commonly Hispanic and less frequently White. However, no clinically meaningful differences were found for patient age, diabetes, CAD, BMI, and HBV core status. Considering the overall high level of missing values among baseline registrant characteristics, no firm conclusions could be made about predisposing factors / conditions for MELD jumps. In multivariable models, adjustment for patient ethnicity, diagnosis at time of registration, serum sodium, and number of records within the past 30 days on the waitlist reduced the magnitude of association of MELD and ΔMELD_30_ with waitlist dropout, though both remained statistically significant.

There are several modeling choices with the ΔMELD that have ramifications for implementation in practice, including decisions about the period of time to evaluate changes and the magnitude of change that is relevant. Our choice of 30-day changes was decided because we felt this represented a manageable time period over which to monitor changes and act on them clinically. Further, since the ΔMELD is inherently noisy minor fluctuations in it should be smoothed or ignored. Here we focused on changes of 10 or more points over 30 days since this had a strong association with waitlist dropout and changes of this magnitude are notable. However, a weakness in this regard is that ΔMELD_30_ ≥ 10 happened relatively infrequently (3.2% of all waitlist observations in our study), especially at MELD scores of 25 or less (Fig 1).

Prior to the switch to MELD-Na, sudden increases in MELD score were associated with increased waitlist dropout, with the biggest discrepancies in the lower MELD range. With the change to MELD-Na this difference is no longer evident, and composite scores combining the MELD and ΔMELD based on data prior to these policy changes over-estimated waitlist dropout associated with MELD jumps at the lower MELD score range. However, the adoption of Share 35 has potentially resulted in comparatively greater waitlist dropout for patients with sharp MELD increases at higher MELD scores (c.f. Fig 6). Our equivalence of two MELD points for ΔMELD_30_ ≥ 10 changes offers a relatively simple rule of thumb for factoring sudden MELD increases into the decision making process for liver transplant registrants at higher MELD scores (35 and above). Another possibility is to use the ΔMELD_30_ as a potential tiebreaker for registrants with identical MELD scores, in lieu of the current policy to prioritize the registrant with the longer waiting time. Positive associations between the ΔMELD_30_ score and transplantation suggests that this reasoning may already be practiced to some extent. This presents an opportunity to extend the benefit of Share 35 to better include those in most critical need.

## Supporting information

S1 Table Correlation between ΔMELD_30_, current MELD, number of measurements, and time between consecutive measurements. Based on UNOS/OPTN data for registrants added to the liver transplant waitlist between 06/30/2003 and 6/30/2013.
(DOCX)
