# Supplementary material for: Use of rapid Model for End-Stage Liver Disease (MELD) increases for liver transplant registrant prioritization after MELD-Na and Share 35, an evaluation using data from the United Network for Organ Sharing
Source: PLoS One. 2019 Oct 3;14(10):e0223053. doi: 10.1371/journal.pone.0223053 (PMC6776460; doi:10.1371/journal.pone.0223053)
Supplement: S1 Table — Based on UNOS/OPTN data for registrants added to the liver transplant waitlist between 06/30/2003 and 6/30/2013. (DOCX) [file pone.0223053.s001.docx]

**S1 Table**: Correlation between ΔMELD_30_, current MELD, number of measurements, and time between consecutive measurements. Based on UNOS/OPTN data for registrants added to the liver transplant waitlist between 06/30/2003 and 6/30/2013.

|  | ΔMELD_30_ | MELD | Number of measurements (30-day window) | Time between consecutive measurements (days) |
| --- | --- | --- | --- | --- |
| ΔMELD_30_ | 1 | 0.56 | 0.33 | -0.19 |
| MELD |  | 1 | 0.45 | -0.44 |
| Number of measurements (30-day window) |  |  | 1 | -0.31 |
| Time between consecutive measurements (days) |  |  |  | 1 |
